# Effects of Melatonin and Epiphyseal Proteins on Fluoride-Induced Adverse Changes in Antioxidant Status of Heart, Liver, and Kidney of Rats

**DOI:** 10.1155/2014/532969

**Published:** 2014-03-26

**Authors:** Vijay K. Bharti, R. S. Srivastava, H. Kumar, S. Bag, A. C. Majumdar, G. Singh, S. R. Pandi-Perumal, Gregory M. Brown

**Affiliations:** ^1^Division of Physiology and Climatology, Indian Veterinary Research Institute (IVRI), Izatnagar, Uttar Pradesh 243122, India; ^2^Nutrition and Toxicology Laboratory, Defence Institute of High Altitude Research (DIHAR), Defence Research and Development Organization (DRDO), Ministry of Defence, C/o- 56 APO, Leh 194101, India; ^3^Division of Animal Reproduction, Indian Veterinary Research Institute (IVRI), Izatnagar, Uttar Pradesh 243122, India; ^4^Somnogen Inc., College Street, Toronto, ON, Canada M6H 1C5; ^5^Department of Psychiatry, Faculty of Medicine, University of Toronto and Centre for Addiction and Mental Health, 250 College Street, Toronto, ON, Canada M5T 1R8

## Abstract

Several experimental and clinical reports indicated the oxidative stress-mediated adverse changes in vital organs of human and animal in fluoride (F) toxicity. Therefore, the present study was undertaken to evaluate the therapeutic effect of buffalo *(Bubalus bubalis)* epiphyseal (pineal) proteins (BEP) and melatonin (MEL) against F-induced oxidative stress in heart, liver, and kidney of experimental adult female rats. To accomplish this experimental objective, twenty-four adult female Wistar rats (123–143 g body weights) were divided into four groups, namely, control, F, F + BEP, and F + MEL and were administered sodium fluoride (NaF, 150 ppm elemental F in drinking water), MEL (10 mg/kg BW, i.p.), and BEP (100 µg/kg BW, i.p.) for 28 days. There were significantly (*P* < 0.05) high levels of lipid peroxidation and catalase and low levels of reduced glutathione, superoxide dismutase, glutathione reductase, and glutathione peroxidase in cardiac, hepatic, and renal tissues of F-treated rats. Administration of BEP and MEL in F-treated rats, however, significantly (*P* < 0.05) attenuated these adverse changes in all the target components of antioxidant defense system of cardiac, hepatic, and renal tissues. The present data suggest that F can induce oxidative stress in liver, heart, and kidney of female rats which may be a mechanism in F toxicity and these adverse effects can be ameliorated by buffalo *(Bubalus bubalis)* epiphyseal proteins and melatonin by upregulation of antioxidant defense system of heart, liver, and kidney of rats.

## 1. Introduction

Various animal diseases and toxicity are often associated with oxidative stress in vital organs, including the kidney, heart, and liver. This pathophysiological stress has multiple effects but is especially characterized by reductions in enzymatic activity, including that of catalase (CAT), superoxide dismutase (SOD), glutathione peroxidase (GPx), and glutathione reductase (GR) [1]. Every trace element is potentially toxic when the rate of exposure is excessive. Increased generation of reactive oxygen species (ROS) is implicated in the pathogenesis of many diseases and in the toxicity of a wide range of compounds, including fluoride (F) [2–4]. Fluoride was found to exert diverse effects on a number of cellular functions and physiological systems, including inhibition of a variety of enzymes [2]. Generation of free radicals, lipid peroxidation, and altered antioxidant defense systems are considered to play an important role in the toxic effects of fluoride [3, 5–7]. Free radicals participate in several reactions, which in turn can produce chain reaction byproducts that also act to damage cells. Oxidative stress results when free radical formation is unbalanced in proportion to the protective antioxidants [8]. Thus, excess free radical formations are associated with many disease states. With increased understanding both of the mechanisms of oxidative stress and the role of antioxidants, it has become apparent that antioxidant defense systems have a balanced coexistence with endogenous reactive oxygen species. Disruption of this balance, such as that which occurs in F toxicity, appears to be one of the major factors producing the oxidative stress associated with diseases involving degeneration of the kidney, heart, and liver [8, 9]. Although evidence regarding the therapeutic value of administering antioxidants in F toxicity is still inconclusive, some studies have nevertheless reported beneficial effects following this strategy [10, 11]. Therefore, evaluation of antioxidant enzymes activities and markers of free radical damage in the heart, liver, and kidney tissue are important for assessing F toxicity. Melatonin is known to have the ability to protect cells from free radical damage [12–14]. Previous studies have shown that melatonin [12, 15] and buffalo epiphyseal proteins (BEP) have various physiological functions that might protect against fluoride and as-induced oxidative stress [16–21]. In view of the cumulative evidence suggesting that antioxidant proteins in the epiphysis could possibly have therapeutic potential, we sought specifically to determine if BEP could protect the kidney, heart, and liver against the F-induced oxidative stress in rats. These findings may have significant implications in elucidating the therapeutic use of buffalo pineal proteins and melatonin as antioxidant drugs in the management of oxidative stress.

## 2. Materials and Methods

The present study was conducted at an altitude of 172 meters above the mean sea level at latitude of 28.20° north and longitude of 79.24° east. All the procedures, conducted on the experimental animals, were duly approved by the Institutional Animal Ethics Committee (IAEC) for the purpose of control and supervision of experiments on animals.

### 2.1. Chemicals

All chemicals used in the study were of analytical grade from HiMedia, Loba Chemie (Mumbai, India); SRL Chemicals, India. Melatonin was procured from Sigma Chemical Co., St. Louis, USA. Buffalo (*Bubalus bubalis*) epiphyseal proteins were supplied by the Neurophysiology Laboratory, Division of Physiology and Climatology, IVRI, Izatnagar.

### 2.2. Experimental Animals

The present study was carried out in adult female Wistar rats of 123–143 g body weights. On arrival at the laboratory all rats were examined for any abnormality or overt signs of ill health. The rats were housed in polypropylene cages and rice husk was used as the nesting material. After a 1-week-long acclimatization period, they were weighed and randomly assigned to four groups so as to give approximately equal initial group mean body weights. Animal room temperature and relative humidity were set at 21 ± 2°C and 50 ± 10%, respectively, and lighting was controlled to give 12 h light and 12 h darkness. All the animals had free access to standard laboratory animal diet and clean water. The animals were checked daily for the health and husbandry conditions.

### 2.3. Experimental Design, Doses, and Mode of Administration of Test Molecules

The experimental design for present study, including group assignment, doses, and route of exposure, is presented in Table 1. BEP and MEL were selected as treatment agents for comparative purposes since we have previously carried out and reported studies which have used these reagents. Appropriate dosages of BEP and MEL were optimized from experience in our previous work and thereafter were dissolved in a suitable vehicle (normal saline-based diluents, pH 7.4) before administration (intraperitoneal route) at exactly 16.00 hrs [18, 20–23]. Melatonin was dissolved in ethanol and further diluted with normal saline. The final concentrations of ethanol in solution were <1%. The F level in drinking water was calculated and thereafter the required concentration of F was made by addition of sodium fluoride daily to drinking water for 28 days [18, 20, 21]. Solutions for administration in experimental animals were prepared daily to minimize possible instability of the chemicals in the mixture. Food intake and water consumption were recorded daily.

### 2.4. Sample Collection

Daily observations were taken for the behavioral changes and mortality, if any, throughout the experimental period. The animals were euthanized, using ether, at the end of the experiment and kidney, heart, and liver were collected immediately. Thereafter, organs were cleaned, rinsed in chilled saline, blotted, and weighed. Then 200 mg of each tissue sample was weighed and placed in 2 mL of ice-cold saline. Another 200 mg of the sample was weighed separately and placed in 2 mL of 0.02 M EDTA for reduced glutathione estimation. Organ homogenates were prepared using an IKA homogenizer (Germany) under ice-cold conditions. Homogenates were collected and centrifuged for 10 min at 3000 rpm. Thereafter, cell free supernatant was collected and transferred to precooled microfuge tubes in duplicate and stored below −20°C. These supernatants were used for estimation of various biochemical parameters to assess body antioxidant defense systems.

### 2.5. Analytical Procedures

Cell free supernatants of tissue homogenates were taken for the analysis of total proteins (organs), lipid peroxidation (LPO), and different parameters of body antioxidant defense systems, in particular, catalase (CAT), superoxide dismutase (SOD), glutathione peroxidase (GPx), and glutathione reductase (GR) activity, as well as nonenzymatic reduced glutathione (GSH) concentration. Lipid peroxidation and GSH in renal, cardiac, and hepatic tissues were measured on the day of tissue collection. Absorbance of all the tissue biochemical estimations was read using Double Beam UV-VIS Spectrophotometer (UV 5704 SS, ECIL, India).

#### 2.5.1. Lipid Peroxidation (LPO)

Lipid peroxidation in tissues sample was determined in terms of malondialdehyde (MDA) production by the method of Rehman [24].

#### 2.5.2. Reduced Glutathione (GSH)

The concentration of GSH in the kidney, heart, and liver was estimated by evaluating free-SH groups and using the DTNB method described by Sedlak and Lindsay [25].

#### 2.5.3. Catalase (CAT)

Activities of catalase enzymes were estimated by spectrophotometry as described by Bergmayer [26].

#### 2.5.4. Superoxide Dismutase (SOD)

Superoxide dismutase activities in liver, heart, and kidney were estimated as per the method described by Madesh and Balasubramanian [27].

#### 2.5.5. Glutathione Peroxidase (GPx)

Glutathione peroxidase activities were determined by the method of Paglia and Valentine [28].

#### 2.5.6. Glutathione Reductase (GR)

This enzyme activity was assayed by the method of Goldberg and Spooner [29].

#### 2.5.7. Protein Assay

Protein contents in liver, heart, and kidney homogenates were determined and calculated by the method of Lowry et al. [30].

#### 2.5.8. Statistical Analysis

Differences between groups were statistically analyzed by a one-way ANOVA, and the differences between the means of groups were separated by the least significant difference (LSD) test. All data were presented as mean ± standard error. Values of *P* < 0.05 were regarded as significant. A computer program (SPSS 10.01, SPSS Inc., Chicago, IL, USA) was used for statistical analysis.

## 3. Results

Cardiac, hepatic, and renal tissues oxidative stress in relation to fluoride treatment and antioxidant properties of melatonin (MEL) and buffalo epiphyseal proteins (BEP) were monitored by the study of products of free radicals such as malondialdehyde (MDA) as well as the activity of enzymes including CAT, GPx, GR, SOD, and GSH levels. Cardiac, hepatic, and renal tissue LPO levels CAT activities were significantly (*P* > 0.05) increased on fluoride administration when compared with the control (Tables 2, 3, and 4). Fluoride treatment resulted in a significant (*P* < 0.05) diminution in the GSH level and activities of cardiac, hepatic, and renal SOD, GR, and GPx (Tables 2, 3, and 4). These data indicate F-induced augmentation of oxidative stress in cardiac, hepatic, and renal tissues.

In F + MEL- and F + BEP-treated animals, MDA (LPO) levels in cardiac, hepatic, and renal tissues were found to be reduced whereas CAT, SOD, GPx, and GR activities and GSH concentration were increased significantly (*P* < 0.05) (Tables 2, 3, and 4). Interestingly, cardiac GR and GSH were higher in F + BEP-treated animals as compared with F + MEL- administered animals (Table 2), whereas higher hepatic SOD activity was observed in F + MEL-administered animals as compared to F + BEP-administered animals (Table 3).

## 4. Discussion

Although some data on F-induced oxidative stress provide supportive evidence for the view that free radicals and oxidative stress are root causes of degenerative disease, there is only a limited amount of animal model data on this point. The present study provides evidence that our in vivo rat model of F-induced oxidative stress is a useful system for studying this pathological condition and in particular for evaluating potential antioxidant therapies such as melatonin and epiphyseal proteins.

Several enzymes are important in antioxidative defense and are basic for their actions in metabolizing either free radicals or reactive oxygen intermediates to no-radical products [31, 32]. Our results suggested that the F treatment depleted antioxidant enzymes and concomitantly increased levels of LPO differentially in rats' liver, heart, and kidney. The findings reported here are consistent with those of others in showing that, in F-treated rats, the combined effect of reductions in antioxidant enzyme activity plus high levels of LPO is associated with deleterious oxidative changes due to the accumulation of toxic products [3, 33, 34]. CAT was the only antioxidant enzyme whose activity was increased. Although GPx and CAT share the substrate H_2_O_2_, the glutathione redox cycle is a major protective mechanism against low levels of oxidant stress, whereas CAT becomes more important in protecting against severe oxidant stress [35]. Guo et al. [2] also reported that antioxidant enzymatic activity as well as overall amounts of antioxidants was reduced in the livers of animals treated with F, conditions which can promote the heavy accumulation of free radicals.

The decrease in the levels of GSH in heart, liver, and kidney observed in our study may have been due to the increased utilization of GSH by GPx in detoxification of H_2_O_2_ generated by F-induced oxidative stress. GSH serves as a reservoir for cysteines which in turn participate in detoxification reactions for xenobiotics and metabolism of numerous cellular compounds [36, 37]. The liver and kidney are the main target organs for attacks by excessive amount of F [38, 39]. Inasmuch as the liver is an important source of GSH, metabolism of xenobiotics in the liver, which can drastically deplete liver GSH, may also result in GSH depletion in other tissues [40]. He et al. [39] also reported that high-fluoride intake enhances oxidative stress in blood and liver, disturbing the antioxidant defense of rats. These events may be implicated in the impaired organ function and poor health of both animals and humans under F-induced oxidative stress.

MEL functions as a free radical scavenger and antioxidant. Its wide range of actions is due in part to its amphiphilicity (it is neutral in cell membranes, i.e., it is neither hydrophobic nor hydrophilic) and thus it easily enters cells and subcellular compartments as well as important morphological barriers such as the blood-brain barrier [41]. In the present study, BEP and MEL reversed the adverse effect of oxidative stress by increasing antioxidant enzyme activity and reducing LPO in cardiac, hepatic, and renal tissues. These findings therefore support the hypothesis that BEP may have antioxidant properties and are also consistent with our earlier findings [18–21]. Additionally, the study demonstrated that the antioxidant activities of BEP were equal in potency and in some cases were superior to those of MEL, which is also produced by the pineal gland. Recently, pineal proteins have been shown to have a role in the stimulation of MEL production [16]. The occasionally superior effects of BEP on MEL may have been due in part to the fact that pineal proteins not only have direct antioxidant effects but also are able to stimulate MEL synthesis, expression of SOD, ceruloplasmin, and other antioxidant enzymes [42–44]. This might have been the reason for the selectively greater antioxidative effect on certain enzymes as compared to MEL action alone. Finally, the findings prove that BEP are effective antioxidants and may play a protective role against cardiac, renal, and hepatic damage induced by oxidative stress following F treatment.

## 5. Conclusions

These findings clearly demonstrate the importance of the pineal gland's neuroendocrine activity and, in particular, of its role as a promoter of antioxidant activity. The study has also elucidated the ameliorative effects of buffalo epiphyseal proteins and melatonin against fluoride-induced oxidative stress. Further, this study findings reveal that exogenous administration of melatonin and buffalo epiphyseal proteins may have therapeutic potential for reducing the fluoride-induced oxidative stress mediated pathogenesis and damage in cardiac, hepatic, and renal damage. However, further study is required to test these molecules in different animals and to know details of these proteins (structure of the individual proteins) with respect to their pharmacological role.

## Figures and Tables

**Table 1:** Distribution of experimental rats to different treatments.

Group	Treatment	Dose	Mode of administration
Control	Normal saline		Intraperitoneal
F	Sodium fluoride + normal saline	150 ppm	Drinking water, intraperitoneal
F + BEP	Sodium fluoride + buffalo epiphyseal proteins (BEP)	150 ppm + 100 *µ*g/kg BW	Drinking water, intraperitoneal
F + MEL	Sodium fluoride + melatonin (MEL)	150 ppm + 10 mg/kg BW	Drinking water, intraperitoneal

**Table 2:** Changes in lipid peroxidation (LPO), catalase (CAT), superoxide dismutase (SOD), glutathione reductase (GR), reduced glutathione (GSH), and glutathione peroxidase (GPx) in heart of female rats (*n* = 6; means ± S.E.).

Group	Parameter					
	LPO (nM MDA/mL)	CAT (nM/min/mg protein)	SOD (U)*	GR (nM/min/mg protein)	GPx (nM/min/mg protein)	GSH (*µ*M/g tissue)
Control	4.63^a^ ± 0.09	141.19^a^ ± 6.05	2.78^b^ ± 0.15	28.33^bc^ ± 1.66	46.40^b^ ± 2.79	6.58^b^ ± 0.20
F	16.24^c^ ± 0.13	201.19^b^ ± 10.52	1.26^a^ ± 0.08	16.92^a^ ± 1.31	30.06^a^ ± 2.21	5.48^a^ ± 0.25
F + BEP	5.66^b^ ± 0.20	152.04^a^ ± 8.81	2.72^b^ ± 0.14	29.96^c^ ± 1.64	54.47^b^ ± 2.76	7.21^c^ ± 0.19
F + MEL	5.03^a^ ± 0.08	151.53^a^ ± 2.27	2.61^b^ ± 0.11	25.98^b^ ± 1.28	50.56^b^ ± 1.53	5.81^a^ ± 0.21

Comparisons are between the rows. Values in the same column bearing no common superscripts (a, b, and c) differ significantly (*P* < 0.05).

*The activity was expressed as U/g of protein [one unit of SOD is the amount (Δg) of protein required to inhibit the MTT reduction by 50%].

**Table 3:** Effect of different treatments on lipid peroxidation (LPO), catalase (CAT), superoxide dismutase (SOD), glutathione peroxidase (GPx), glutathione reductase (GR), and reduced glutathione (GSH) in liver of female rats (*n* = 6; means ± S.E.).

Group	Parameter					
	LPO (nM MDA/mL)	CAT (nM/min/mg protein)	SOD (U)*	GR (nM/min/mg protein)	GPx (nM/min/mg protein)	GSH (µM/g tissue)
Control	4.61^a^ ± 0.11	291.03^a^ ± 14.93	6.15^b^ ± 0.37	145.85^c^ ± 7.33	25.67^b^ ± 1.87	3.30^b^ ± 0.06
F	12.21^c^ ± 0.77	385.63^b^ ± 13.50	2.58^a^ ± 0.16	83.92^a^ ± 10.10	14.87^a^ ± 0.91	2.53^a^ ± 0.14
F + BEP	6.33^b^ ± 0.11	276.36^a^ ± 12.12	5.53^b^ ± 0.21	119.33^b^ ± 7.69	25.88^b^ ± 1.28	3.29^b^ ± 0.06
F + MEL	6.28^b^ ± 0.11	269.54^a^ ± 14.38	7.81^c^ ± 0.18	109.73^b^ ± 7.82	27.14^b^ ± 0.90	3.15^b^ ± 0.10

Comparisons are between the rows. Values in the same column bearing no common superscripts (a, b, and c) differ significantly (*P* < 0.05).

*The activity was expressed as U/g of protein [one unit of SOD is the amount (Δg) of protein required to inhibit the MTT reduction by 50%].

**Table 4:** Effect of different treatments on lipid peroxidation (LPO), catalase (CAT), superoxide dismutase (SOD), glutathione peroxidase (GPx), glutathione reductase (GR), and reduced glutathione (GSH) in kidney of female rats (*n* = 6; means ± S.E.).

Group	Parameter					
	LPO (nM MDA/mL)	CAT (nM/min/mg protein)	SOD (U)*	GR (nM/min/mg protein)	GPx (nM/min/mg protein)	GSH (*µ*M/g tissue)
Control	4.99^a^ ± 0.130	251.70^a^ ± 4.58	5.98^b^ ± 0.17	126.64^c^ ± 8.47	20.13^b^ ± 1.11	3.50^b^ ± 0.06
F	16.62^c^ ± 0.360	382.46^c^ ± 14.89	2.56^a^ ± 0.12	41.11^a^ ± 5.40	14.38^a^ ± 0.49	2.68^a^ ± 0.09
F + BEP	6.90^b^ ± 0.140	295.11^b^ ± 4.22	5.64^b^ ± 0.37	85.85^b^ ± 3.97	20.18^b^ ± 2.59	3.76^b^ ± 0.08
F + MEL	6.88^b^ ± 0.089	307.06^b^ ± 5.24	5.96^b^ ± 0.28	93.01^b^ ± 7.78	18.43^b^ ± 0.83	3.25^b^ ± 0.10

Comparisons are between the rows. Values in the same column bearing no common superscripts (a, b, and c) differ significantly (*P* < 0.05).

*The activity was expressed as U/g of protein [one unit of SOD is the amount (Δg) of protein required to inhibit the MTT reduction by 50%].
